# 

**Published:** 1967-01

**Authors:** 


### *Contents*

| | | Page |
|---|---|---|
| ON LISTENING TO THE NECK ........................ | *T. W. Lloyd* | 1 |
| DIPYRIMADOLE & POST-OPERATIVE HYPERCOAGULABILITY .................................................. | *U. Jayaswal* | 8 |
| AN APPROACH TO THE ANALYSIS OF MENORRHAGIA | *P. F. Fairbrother* | 11 |
| CARCINOMA OF THE LUNG WITH CUSHING'S SYNDROME: A Clinico-Pathological Conference | | 15 |

